# Synergistic Activity of Econazole-Nitrate and Chelerythrine against Clinical Isolates of Candida albicans

**Published:** 2014

**Authors:** Zhibao Chen, Xinran Li, Xiuping Wu, Wei Wang, Wendong Wang, Mingxun Xin, Fengge Shen, Lihui Liu, Junchao Liang, Lei Li, Lu Yu

**Affiliations:** a College of Life Science and Technology, Heilongjiang Bayi Agricultural University, Daqing, Heilongjiang 163319, China.; b Key Laboratory of Zoonosis Research, Ministry of Education, Institute of Zoonosis, College of Animal Science and Veterinary Medicine, Jilin University, Changchun, China.

**Keywords:** Candida albicans, Synergism, Econazole-nitrate, Chelerythrine

## Abstract

The aim of this investigation was to assess the *in-vitro* interaction of two antifungal agents, econazole-nitrate and chelerythrine, against ten fluconazole-resistant clinical isolates and one ATCC type strain 10231 of *Candida albicans*. The checkerboard microdilution method was performed according to the recommendations of the National Committee for Clinical Laboratory Standards, and the results were determined by visual examination. The interaction intensity was tested in all isolates using the fractional inhibitory concentration index (FICI). These experiments showed synergism between econazole-nitrate and chelerythrine in antifungal activity against *C. albicans*, and no antagonistic activity was observed in any of the strains tested. Moreover, time-kill curves were performed with selected strains to confirm the positive interactions. The similarity between the results of the FICI values and the time-kill curves revealed that chelerythrine greatly enhances the antifungal effects of econazole-nitrate against isolates of *C. albicans*. This synergistic effect may markedly reduce the dose of econazole-nitrate required to treat candidiasis, thereby decreasing the econazole-nitrate toxic side effects. This novel synergism might provide a potential combination treatment against fungal infections.

## Introduction

The dimorphic fungus,* Candida albicans,* is a major fungal pathogen responsible for causing a variety of candidiasis. It is the fourth leading cause of nosocomial infections, with a mortality rate approaching 50% (1,2). The organism is known to cause local infections, such as vaginitis and thrush, and can cause serious life-threatening invasive and systemic disease, especially among immunocompromised and immunodeficient patients who are receiving broad-spectrum antibiotics, patients undergoing cancer chemotherapy, organ transplant recipients, or individuals infected with the human immunodeficiency virus (HIV) (3,4).

Currently, the azoles, which have a broad-spectrum antifungal activity against a wide variety of candida species, are widely used for both the prevention and treatment of candidiasis (5). However, more recently, the treatment of candidal infections has led to several problems. In addition to the toxicity presented by some fungicidal agents, such as amphotericin B (AMB)(6), other conventional fungistatics have been rendered ineffective by resistance or dose-dependent susceptibility found in some* C. albicans i*solates. In particular, azole-resistant isolates are appearing at a high frequency due to the increasing clinical use of the azoles. Thus, new therapeutic strategies to cope with candidal infections are necessary. Combination therapy is a novel approach that can be used to decrease the toxicity of an antifungal drug and improve the efficacy of the antifungal therapy and may be especially useful for treating infections caused by drug-resistant fungi (7).

**Figure 1:**
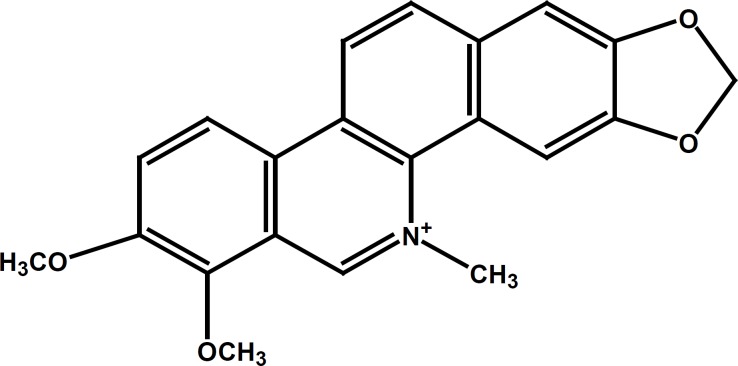
Chemical structure of Chelerythrine. (Figure composed using Chem Draw 7.0).

Chelerythrine (CHT, C_21_H_17_NO_4_, as illustrated in Figure 1), one of the more important benzophenanthridine alkaloids derived from the roots of Chelidonium majus, has been shown to have various biological activities, including antimicrobial, antiplatelet, and antitumor activities (8,9,10). Additionally, a previous report showed that CHT has been recommended for medical use in the treatment of oral inflammatory processes due to its low toxicity and strong anti-inflammatory effects (11, 12). 

In this study, we investigated the antifungal activity of CHT and econazole-nitrate (ECZN) against *C. albicans*, and assessed the combination of CHT and ECZN for the treatment of candidasis. To evaluate this combination for synergism, the checkerboard microtiter test and time-kill assays were performed.

## Experimental

*Strains and growth conditions*

*C. albicans* ATCC 10231 was obtained from the American Type Culture Collection (ATCC, Gaithersburg, MD, USA). In addition, the ten fluconazole-resistant isolates of *C. albicans* used in this study were kindly provided by Jiang Y.Y. The strains were maintained on Sabouraud dextrose agar (SDA, 4% glucose, 1% Bacto peptone and containing 3% agar) plates and stored at 4 ℃ during the experimental period. *C. albicans* ATCC 10231 was used as the quality control strain.

*Antifungal agents*

CHT and ECZN were used in this study. The CHT (≥98% pure) and the ECZN (≥98% pure) were purchased from the National Institute for the Control of Pharmaceutical and Biological Products, Beijing, China. DMSO (dimethyl sulfoxide) was used to prepare stock solutions of CHT (20480 µg/mL) and ECZN (40960 µg/mL). All of the antifungal stock solutions were maintained at -20 ℃. The final concentration of DMSO in the wells was less then 1% v/v, which did not affect the growth of the test organisms in all of the susceptibility tests (13).

*Antifungal susceptibility testing*

The minimum inhibitory concentrations (MICs) of CHT and ECZN against the Candida strains were determined using the broth microdilution method as described by the Clinical and Laboratory Standards Institute (CLSI, formerly the National Committee for Clinical Laboratory Standards) document M27-A. The susceptibility test was performed in a 96-well flat-bottomed microtitration plate according to the process of L. Drago *et al*. (14). Briefly, all tested isolates were incubated at 35 ℃ in Sabouraud dextrose broth (SDB and diluted with the same fresh medium to a density of ~10^6 ^cfu/mL, which was further diluted to generate a final concentration of 5×10^5 ^cfu/mL dilutions. The MIC values of CHT and ECZN were tested after serial 2-fold dilutions in 96-well flat-bottomed microtitration plates in SDB, and the final concentrations of the antimicrobial broth ranged from 0.25 to 512 µg/mL. The MIC was defined as the lowest concentration with no visible growth compared to that of the drug-free control. The quality control (QC) strain, *C. albicans* ATCC 10231, was included in each batch of the susceptibility tests to ensure quality. 

*Checkerboard* *method*

Interactions between ECZN and CHT were tested in 96-well flat-bottomed microtitration plates by the checkerboard method against the ten *C. albicans* isolates and the susceptible *C. albicans* ATCC 10231 strain in the same medium as used previously. The final antimicrobial agent concentrations after the addition of 100 µL of inoculum ranged from 0.25 µg/mL to 512 µg/mL for ECZN and from 8 µg/mL to 512 µg/mL for CHT. The inocula were prepared at a final concentration of 5×10^5 ^cfu/mL per well. The plates were incubated at 35 ℃ for 24 - 48 h. The effects of the combinations of antimicrobial agents were interpreted by the fractional inhibitory concentration index (FICI). Based on LA theory, the FICI was calculated by the following equation (15):

FICI = FICA + FICB = MIC_A_^comb^ ⁄ MIC_A_^alone^ + MIC_B_^comb^ ⁄ MIC_B_^alone^

The effect of the combinations of antimicrobial agents was classified by the following standard: (1) FICI ≤ 0.5, synergistic effect; (2) 0.5 ≤ FICI ≤ 4.0, additive or indifferent; and (3) FICI > 4.0, antagonistic. Student’s t-test analysis was performed to analyse the means of MICs between used ECZN alone and the ECZN-CHT combination, based on spss version 17.0 for windows. p-values<0.05 were accepted as statistically significant.

*Time-kill studies*

Time–kill studies were performed with the chosen isolates using the methodology of L. Drago *et al.* (14). DMSO comprised <1% of the total test volume. CHT and ECZN were diluted in SDB to obtain a final concentration of 1/2 MIC. *C. albicans* 687 and 762 were prepared at the starting inoculum of 10^6 ^cfu/mL of 0.5 mL volume to obtain a final concentration of 10^5 ^cfu/mL in the 5 mL final volume system. The concentrations of the agents were determined by the MIC values obtained in the previous experiment. The tubes containing CHT (32 µg/mL), ECZN (16 µg/mL), CHT/ECZN (32 µg/mL and 16 µg/mL, respectively) and 10^5 ^cfu/mL of the tested isolates were incubated at 35 °C. At various predetermined time points (0, 12, 24, and 48 h), 100 µL aliquots were removed from each test tube and serially diluted 10 fold in sterile water. A volume of 100 µL of each dilution was spread on the Sabouraud dextrose agar plates to incubate at 35 °C for 24 h prior to colony counts enumeration. Each assay was performed in triplicate. Synergism and antagonism were defined by the following criteria (16): (1) synergy: a 2 log10cfu/mL decrease by the combination compared to the most active agent; (2) antagonism: a 2 log10cfu/mL increase by the combination compared to the most active agent; and (3) indifferent: a change of < 2 log10cfu/mL between the combination and the most active agent.

## Results

*Antifungal activities and interactions of drugs*

The *in-vitro* antifungal activities of CHT and ECZN alone and in combination were assessed. The results for the tested drugs alone and the checkerboard analysis are summarized in Table 1. 

In testing the two antifungals independently, the MIC values for these two agents against the clinical isolates of *C. albicans* ranged from 16 to 32 µg/mL for ECZN and 32 to 128 µg/mL for CHT treatment. These results showed that CHT has antifungal activity against clinical isolates of *C. albicans* *in-vitro*. 

In the combination studies, the interaction between ECZN and CHT displayed synergism for all tested strains, including *C. albicans* ATCC 10231, with FICI values ranging from 0.078125 to 0.5 using the FICI method. Moreover, an antagonism interaction was not observed for any of the tested strains. As shown in Table 1, the ECZN-CHT combination markedly reduced the MICs. The data above showed that there was a good synergistic antifungal effect against *C. albicans* when CHT was combined with ECZN.

**Table 1:** *In* *-* *vitro* interaction between chelerythrine and econazole-nitrate against clinical isolates of *Candida albicans*

**Strains**	**Median MIC (range) of durg along (µg/mL)**		**Median MIC (range) in combination (µg/mL)**		**Results**	
	**CHT**	**ECZN**	**CHT**	**ECZN**	**FICI**	**INT**
*C. albicans* 580	32	16	8	1	0.3125	SYN
*C. albicans* 659	64	16	4	1	0.125	SYN
*C. albicans* 687	64	32	16	8	0.5	SYN
*C. albicans* 762	64	32	16	4	0.375	SYN
*C. albicans *817	128	32	16	1	0.15625	SYN
*C. albicans* 876	128	32	8	0.5	0.078125	SYN
*C. albicans* 885	64	16	16	2	0.375	SYN
*C. albicans* 893	32	32	8	0.5	0.265625	SYN
*C. albicans* 904	64	32	16	4	0.375	SYN
*C. albicans* 0604109	64	32	16	4	0.375	SYN
*C. albicans* 10231	128	32	16	2	0.1875	SYN

^Note:^ ^CHT, chelerythrine; ECZN, econazole-nitrate; MIC,minimum inhibitory concentration; FICI, fractional inhibitory concentration index; INT, interpretation; SYN, synergism; ADD, additive; IND, indifference; ANT, antagonism. There is a significant reduced between used ECZN alone and the ECZN-CHT combination by Student^ ^t-^ ^test (P<0.05).^

*Time-kill curves*

**Figure 2:**
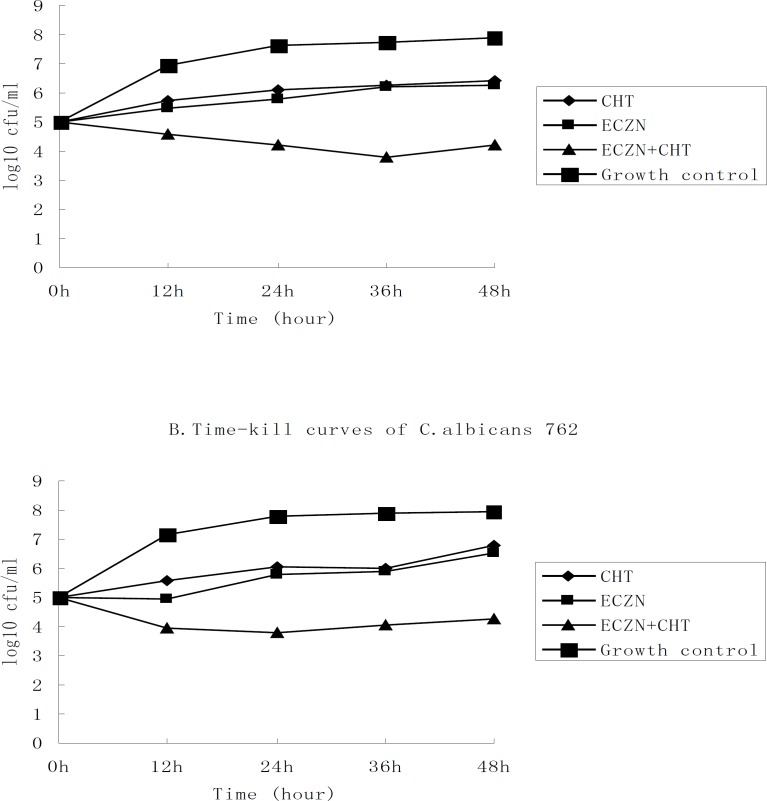
Time–kill assays with econazole-nitrate and chelerythrine alone and in combination against two species of clinical C. albicans (687 and 762). The starting inoculum density of the strains was 10^5^ cfu/mL. The concentrations of antimicrobial were 32 µg/mL for CHT and 16 µg/mL for ECZN. And at the predetermined time points (o h, 12 h, 24 h, 36 h, 48 h), the bactericidal activity of the compounds were examined. Bacterial counts are represented as log 10cfu/mL

To analyze the interaction of these drug combinations, we used the time-kill approach. The results of the time-kill curves with the antimicrobials alone or in combination against two chosen clinical isolates, *C. albicans* 687 and 762, are presented in Figure 2. As shown in the graph, the lines that represent the agents alone have a similar tend. This suggests that the tested strains had similar susceptibilities to CHT (32 µg/mL) and ECZN (16 µg/mL). Given an initial inoculum density of 10^5^cfu/mL, combination therapy yielded a 2.06 log10cfu/mL decrease for *C. albicans* 687 and a 2.26 log10cfu/mL decrease for *C. albicans* 762, compared to 16 µg/mL of ECZN after 48 h of incubation. The fungistatic activity of ECZN was dramatically enhanced by the addition of CHT. For the two strains tested, time–kill curves verified synergism for the ECZN⁄CHT combination.

## Discussion

A synergistic strategy can be an important approach for the treatment of disease, as it often shows a better effectiveness compared to monotherapy and can lower drug dosage requirements, reduce the toxic side-effects of drugs and prevent or delay the emergence of drug resistance. Recently, there have been many reports that have shown synergistic effects between antibacterial agents or peptides in combination with fluconazole against *C. albicans *(17,18,19 and 20), but few studies have been conducted investigating synergism with ECZN. ECZN, which belongs to the imidazoles, is another important antifungal agent largely used for the treatment of many nosomycosis, especially surface infections, such as mucous membranes infections, dermatophytosis and vaginitis (21). The present study was undertaken to analyze the drug–drug interactions between ECZN and CHT using the checkerboard microdilution method. Based on the experimental data, it can be concluded that a synergistic combination effect was observed in all of the test strains, and no antagonistic action was observed. Furthermore, the positive interactions between the two antimicrobials were also confirmed by the time–kill curves in the selected strains. Moreover, the results of both indicated that there was good agreement between the conclusions drawn from the FICI method and the time–kill curves for the strains tested.

In *C. albicans*, there are four targets (cell wall biosynthesis, membrane integrity, sterol biosynthesis and DNA/RNA synthesis) of fungicidal agents. The specific mechanism of the antifungal effect of ECZN has been reported. In brief, ECZN inhibits the activity of the cytochrome P-450 lanosterol 14a-demethylase, which plays important roles in the ergosterol biosynthetic pathway, and this pathway is considered to be the primary target of the azole antifungal drugs (22,23). Hence, two effects are produced. First, it disrupts the biosynthesis of ergosterol (the main sterol in the fungal cell membrane), which influences cell membrane integrity. Second, it can accumulate toxic methylated sterol intermediates that can damage the fungal cell. 

Although the underlying mechanism of the synergism between ECZN and CHT remains unclear, some understanding may be derived from previous studies. CHT has been reported to have significant antibacterial activity against Gram-positive bacteria and *C. albicans *(24) and has recently been extensively studied as a protein kinase C (PKC) inhibitor (25,26,27). Its antimicrobial activity may be involved with the inhibitory action against PKC. PKC has been associated with the regulation of cell proliferation, differentiation, and survival, and it has been widely studied in fungi. More recently, many researchers have shown that pkc1, a more primitive PKC isoenzyme in fungus, can regulate chitin (a component of the cell wall) synthesis and drug susceptibility in *C. albicans*. The inhibition of pkc1 could enhance the efficacy of antifungal agent targeting the cell membrane, including the azoles (2,28). Thus, it can be inferred that the mechanism of synergy between ECZN and CHT may be due to the PKC inhibitory effect of CHT. In addition, recent studies have shown that both CHT and ECZN produce reactive oxygen species (ROS), which leads to apoptosis (29,30,31). Moreover, CHT has been shown to play a role in DNA damage (32). Although there is no evidence to indicate that all of these drug characters could act on *C. albicans*, hypothetically, these functions may be involved in the synergic mechanism. The exact cooperative mechanism may be multifactorial and will have to be further explored.

CHT is a potent PKC inhibitor and antifungal compound that may have more significant therapeutic potential against candidiasis, especially in immunocompromised patients, due to its low toxicity and antitumor activity. In conclusion, the results of this study suggest that CHT can markedly enhance the effects of ECZN against isolates of *C. albicans*. Moreover, this synergism can markedly reduce the dosage requirements of ECZN, decreasing the ECZN toxic side effects. This ECZN-CHT synergism may have significant clinical implications for the treatment of superficial mycosis.
